# Integrated lung ultrasound score for early clinical decision-making in patients with COVID-19: results and implications

**DOI:** 10.1186/s13089-022-00264-8

**Published:** 2022-06-01

**Authors:** Paola Dell’Aquila, Pasquale Raimondo, Vito Racanelli, Paola De Luca, Sandra De Matteis, Antonella Pistone, Rosa Melodia, Lucilla Crudele, Daniela Lomazzo, Antonio Giovanni Solimando, Antonio Moschetta, Angelo Vacca, Salvatore Grasso, Vito Procacci, Daniele Orso, Luigi Vetrugno

**Affiliations:** 1 Emergency Department, Teaching Hospital Policlinico di Bari, Bari, Italy; 2 Department of Emergency and Organ Transplant, University of Bari Aldo Moro, Bari, Italy; 3 Department of Biomedical Sciences and Human Oncology, Section of Internal Medicine “Guido Baccelli, University of Bari Medical School, Bari, Italy; 4 Department of Interdisciplinary Medicine, University of Bari “Aldo Moro”, Bari, Italy; 5 Department of Anesthesia and Intensive Care Medicine, ASUFC Hospital of Udine, Udine, Italy; 6 Department of Medical, Oral and Biotechnological Sciences, University of Chieti-Pescara, Chieti, Italy

**Keywords:** COVID-19, SARS-CoV-2, Lung Ultrasound Score (LUS), Integrated Lung Ultrasound Score (i-LUS), Emergency Department

## Abstract

**Background and objectives:**

Lung Ultrasound Score (LUS) identifies and monitors pneumonia by assigning increasing scores. However, it does not include parameters, such as inferior vena cava (IVC) diameter and index of collapse, diaphragmatic excursions and search for pleural and pericardial effusions. Therefore, we propose a new improved scoring system, termed “integrated” lung ultrasound score (i-LUS) which incorporates previously mentioned parameters that can help in prediction of disease severity and survival, choice of oxygenation mode/ventilation and assignment to subsequent areas of care in patients with COVID-19 pneumonia.

**Methods:**

Upon admission at the sub-intensive section of the emergency medical department (SEMD), 143 consecutively examined COVID-19 patients underwent i-LUS together with all other routine analysis. A database for anamnestic information, laboratory data, gas analysis and i-LUS parameters was created and analyzed.

**Results:**

Of 143 enrolled patients, 59.4% were male (mean age 71 years) and 40.6% female. (mean age 79 years: *p* = 0.005). Patients that survived at 1 month had i-LUS score of 16, which was lower than that of non-survivors (median 20; *p* = 0.005). Survivors had a higher PaO2/FiO2 (median 321.5) compared to non-survivors (median 229, *p* < 0.001). There was a correlation between i-LUS and PaO2/FiO2 ratio (rho:-0.4452; *p* < 0.001), PaO2/FiO2 and survival status (rho:-0.3452; p < 0.001), as well as i-LUS score and disease outcome (rho:0.24; *p* = 0.005). In non-survivors, the serum values of different significant COVID indicators were severely expressed. The i-LUS score was higher (median 20) in patients who required non-invasive ventilation (NIV) than in those treated only by oxygen therapy (median 15.42; *p* = 0.003). The odds ratio for death outcome was 1.08 (confidence interval 1.02–1.15) for each point increased. At 1-month follow-up, 65 patients (45.5%) died and 78 (54.5%) survived. Patients admitted to the high critical ward had higher i-LUS score than those admitted to the low critical one (*p* < 0.003).

**Conclusions:**

i-LUS could be used as a helpful clinical tool for early decision-making in patients with COVID-19 pneumonia.

**Supplementary Information:**

The online version contains supplementary material available at 10.1186/s13089-022-00264-8.

## Introduction

Lung ultrasound’s crucial role in diagnosing and monitoring of COVID-19 interstitial pneumonia, has been highlighted during this pandemic surge [1–3]. The good new is that lung ultrasound signs in COVID-19 remain comparable to other interstitial pneumonia [4–6], separate B-lines, coalescent B-lines, confluent B-lines, subpleural consolidation and irregular pleural line [7]. In COVID-19, Volpicelli et al. [8] reported a new lung ultrasound sign, termed as the “light beam” probably corresponding to the “ground glass” opacity detected by computed tomography (CT) scan in a very early stage [8]. Although all these signs are not pathognomonic of for COVID-19 interstitial pneumonia, they assumed a high positive predictive value, with a high probability for correct diagnosis during the pandemic surge [1, 6, 9]. The lung ultrasound score (LUS), which measure the severity of the superficial lung disease [8], has been shown to predict disease outcomes and evolution of interstitial pneumonia in intensive care patients over time [9, 10]. However, LUS evaluation does not include important parameters, such as pleural line characteristics [11, 12], COVID-19 cardiac involvement, such as pericardial effusion [13, 14] or the volemic status and intrathoracic resistances through the inferior vena cava (IVC) diameter evaluation [15, 16], and the diaphragm load [17]. Therefore, we hypothesized that a broader score which integrates all these aspects too (Integrated Lung Ultrasound Score, i-LUS), could help clinicians in the rapid management of patients with COVID-19 interstitial pneumonia admitted to our sub-intensive section of the Emergency Medicine Department (SEMD). The aim of the work was to evaluate the role of i-LUS in the urgent clinical decision-making process as a tool that can help to stratify the severity of patients and support the oxygenation/ventilation/fluid management, including the patient’s adequate allocation to the care department. We also discuss the other COVID-19 disease related data, such as comorbidities, sex and patients’ age, which we observed during the investigation.

## Materials and methods

### Study population

This prospective study was approved by our Institutional Review Board, with the approval number #6524—Ethics Committee—Policlinico di Bari. In the period from March 8 to April 15, 2020, 143 consecutive patients with positive SARS-CoV-2 molecular test (nasopharyngeal swab), symptoms of dyspnea, O2 saturation values less than 92%, with or without fever, and hypotension (as inclusion criteria), were enrolled. Exclusion criteria were: patients with signs and symptoms of pneumonia but negative SARS-CoV-2 molecular test, problematic ultrasonographic window, known pulmonary autoimmune diseases, or refusal to participate in the study.

### Study protocol

**Fig. 1:**
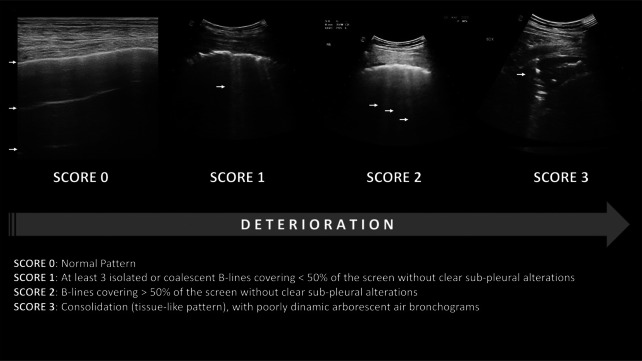
Lung ultrasound score

In compliance with the highest level of personal protective equipment of the World Federation for Ultrasound in Medicine and Biology (WFUMB), all the bedside lung ultrasound examinations were performed by experienced physician. Within one hour of admission, a complete lung ultrasound evaluation was performed according to the LUS score (Fig. 1), (see Additional file 1). All measurements were obtained with an Esaote My Lab 70 Gold ultrasound system with 2.5–5 MHz convex probes, 7–12 MHz linear and 2.5 MHz sectorial probes. The external lung fields were examined by longitudinal and transverse plane scans, dividing the surface of the thorax into 12 zones: 6 on the right (anterior: upper R1 and lower R2; lateral; upper R3 and lower R4; posterior: upper R5 and lower R6) and 6 on the left side (front: upper L1 and lower L2; lateral: upper L3 and lower L4; rear: upper L5 and lower L6). LUS assigns 0 points to A lines or < 2 separate B lines plus regular sliding; 1 point with lines B ≥ 3 or spaced focal points plus regular sliding; 2 points with coalescing B lines, and 3 points to pulmonary consolidations with a score ranging from 0 (normal lungs) to 36 (worst case scenario). LUS evaluation has been subsequently integrated with following four additional parameters [6]:

- Presence of pleural effusion (cm) (value 0 absent, value 1 present);
- Presence of pericardial effusions (cm) (value 0 absent, value 1 present);
- Measurement of the IVC respiratory variation (< 0–33%) (value 0 absent, value 1 present);
- Diaphragm excursion (cm) [18, 19]. This last parameter was measured during the normal breathing (in O2 therapy or before non-invasive ventilation, NIV), in M-mode through a right subcostal scan. An excursion > 2 ± 0.5 cm was considered as normal (value 0, absent) while the value below as abnormal (value 1, present).

**Fig. 2:**
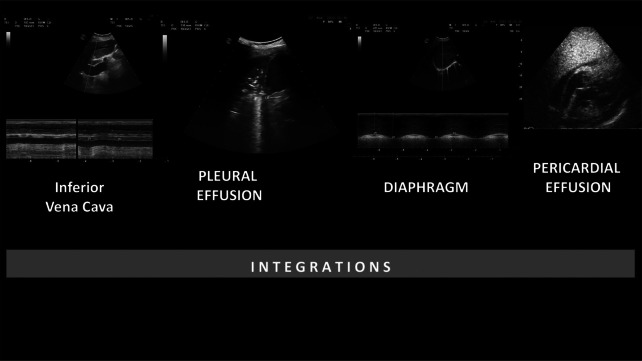
Integrations

The elements reported above were integrated with the LUS score to provide i-LUS (Fig. 2). Thus, the total value of the score was increased from 36 to 40 points.

### Statistical analysis

Each variable was collected on a datasheet (Microsoft Excel for Mac). To summarize data, a descriptive statistic was used. Data were reported as medians and interquartile ranges or means and standard deviations, as appropriate. Categorical variables were summarized as counts and percentages. No imputation was made for missing data. The normality of the distribution of the variables was assessed using Shapiro Test. The Wilcoxon rank-sum test performed median comparisons for baseline parameters between two groups, whereas the Kruskal–Wallis test was used for median comparison among more groups. Spearman's correlation coefficient performed correlation analysis. A *P* value < 0.05 was considered statistically significant. A logistic regression model was developed to assess the Odds Ratio for mortality. Mac's statistical analysis was performed with R Studio, version 1.2.5042 (R Project for Statistical Computing).

## Results

### Patient outcomes

Out of 143 enrolled patients, 85 were male (59.4%) and 58 female (40.6%). The mean age was 71.5 ± 14.9 years, with a median of 73 years. Males had a median age of 71 years (interquartile range 59–80 years), while females 79 (interquartile range 59–80 years; *p* = 0.005). The younger patient was 27 years while the older one 99, both male. Table 1 shows the list of comorbidities according to the main outcome of interest at the end of the follow-up: 43.4% of patients had another concomitant disease upon admission, 30% had two diseases, and 25.9% more than two.

**Table 1:** Demographic characteristics and coexisting conditions among COVID-19 disease non-survivors and survivors

	Dead (65 pts)	Alive (78 pts)	*P* value
Age (Years: Mean ± SD)	78.6 (± 11,3)	65.5 (± 15)	0.006*
Male (Nr, %)	37 (56.9%)	48 (61.5%)	0.60**
Female (Nr, %)	28 (43.1%)	30 (38.4%)	0.60**
Hypertension (Nr, %)	36 (55.4%)	34 (43.6%)	0.39**
Obesity (Nr, %)	10 (15.4%)	9 (11.5%)	0.60**
COPD (Nr, %)	20 (30.7%)	10 (12.8%)	0.02**
Diabetes (Nr, %)	20 (30.7%)	11 (14.1%)	0.04**
Neuropsych. Pathol. (Nr, %)	27 (41.5%)	16 (20.5%)	0.02**
Heart disease (Nr, %)	29 (44.6%)	18 (23%)	0.02**
Neoplasia (Nr, %)	7 (10.7%)	8 (10.3%)	0.99**

^*^T Test

^**^Fisher Test

### i-LUS results

**Fig. 3:**
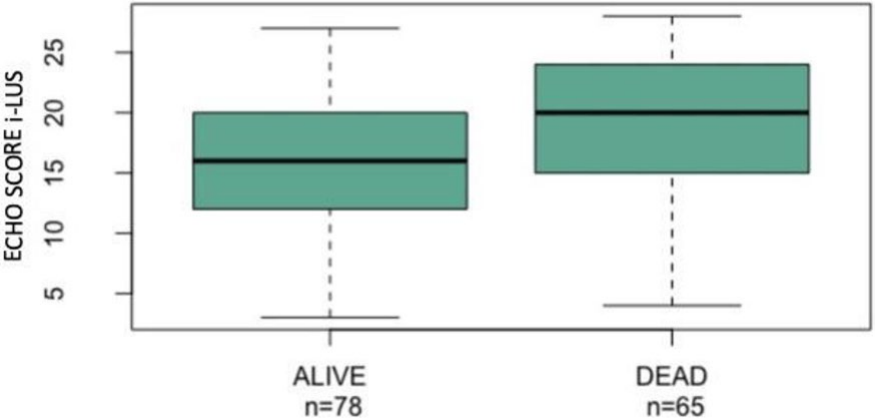
Boxplots—Echo Score i-LUS (i-LUS) and survival (*p* value = 0.005136)

In the survivor group, patients had a median i-LUS score of 16 (interquartile range 12–20), while the score was 20 in the non-survivor group (interquartile range 15–24; *p* = 0.005; Fig. 3). Patients which survived had a higher PaO2/FiO2 ratio on admission (median 321.5; interquartile range 249.7–394.7) than those who died (median 229; interquartile range 123–324; *p* < 0.001). The i-LUS and PaO2/FiO2 ratio were significantly correlated (rho: − 0.4452; *p* < 0.001), as were PaO2/FiO2 ratio and the survival status (rho: − 0.3452; p < 0.001). The i-LUS analysis of diaphragm excursion was extrapolated, showing a median excursion in surviving patients of 20 mm (interquartile range 13–23), whereas it was 16 mm in the deceased ones (interquartile range 12–21, *p* = 0.17). Furthermore, a significant correlation between i-LUS and the disease outcome (rho: 0.24; *p* = 0.005) as well as i-LUS and diaphragm excursion (rho: − 0.45; *p* < 0.001] were noted. The surviving patients had lower indices of LDH, CPK, CRP, lactates, myoglobin, troponin, presepsin and D-dimers on laboratory tests compared with deceased patients, as shown in Additional file 2: Table S1. The CRP values also correlated with i-LUS (rho: 0.3243; *p* < 0.001) and PaO2/FiO2 (rho: − 0.2871; *p* < 0.001).

**Fig. 4:**
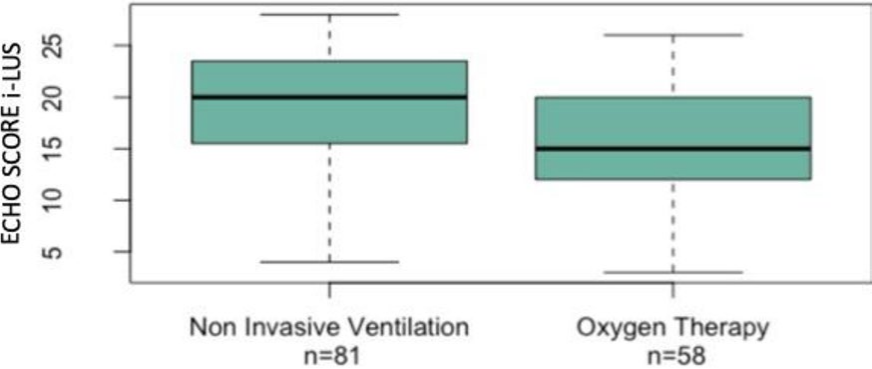
Boxplot—ECHO SCORE i-LUS and support of the respiratory function

**Fig. 5:**
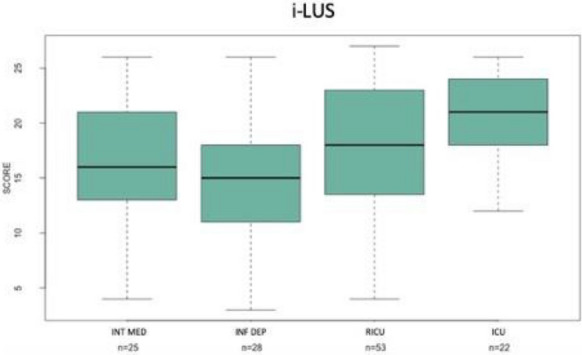
Boxplots—i-LUS and hospitalization areas: *INT MED* Internal Medicine, *INF DEP* Infectious Diseases Department, *RICU* Respiratory Intensive Care Unit, *ICU* Intensive Care Unit

All studied patients had an average length of stay in SEMD from 24 to 72 h and, during the stay, they received oxygen therapy or NIV. i-LUS was significantly higher in patients which required NIV (median 20) than those treated by oxygen therapy only (median 15.42; *p* = 0.003; Fig. 4). Fourteen patients, out of 143, died during their stay at SEMD. For the remaining 129, Fig. 5 shows the median i-LUS value and hospitalization areas, stratified according to the ward of destination (pairwise comparisons Kruskal–Wallis rank-sum test *p* value = 0.003). The mortality at 30 days from access was 45.5% (65 patients died and 78 survived). Thirty-seven male (43%) died at follow-up, while 48 survived (57%). Twenty-eight (48%) female died at follow-up, while 30 (52%) survived (*p* = 0.7). The odds ratio for death was 1.08 (confidence interval 1.02–1.15) for each one-point i-LUS value increase. In the multivariate analysis considering echo score, age and gender, the odds ratio of the echo score, for death was 1.08 (confidence interval 1.02–1.15) for each point increased, for age 1.08 (confidence interval 1.04–1.13), that for male gender 1.6 (confidence interval 1.06–3.15). Adding comorbidities (Age, Diabetes, Hypertension, Obesity, COPD, Neuropsychiatric pathology, Heart disease, Neoplasia) resulted in only age and diabetes retaining statistical significance (see Additional file 1).

## Discussion

Since early 2020, the use of LUS in COVID-19 patients has received much attention from clinicians, since this procedure can help them to promptly identify and classify disease severity [1–3]. Indeed, a high score is related to worsening pulmonary aeration [20, 21]. Although none of the LUS characteristics is pathognomonic for COVID-19, many evidences support its value, and a new definition of “light beam” and “waterfalladdit sign has been proposed to increase diagnostic accuracy [7, 8, 17]. During the pandemic, LUS was subjected to various implementations altogether with clinical or morphological parameters. Some authors have emphasized the role of thoracic ultrasound integrated with echocardiography to improve the evaluation of patients with COVID-19 pneumonia in the suspicion of myocarditis, ventricular dysfunction and ventricular thrombi [22]. Some authors have correlated LUS ultrasound probability models with clinical phenotypes to anticipate the diagnosis of COVID pneumonia 19 [8]. Others have shown that LUS must be complemented by the investigation of potential cardiovascular abnormalities, especially in patients undergoing invasive and non-invasive ventilation [23].

In this study, we associate the standard two-dimensional LUS with other ultrasound achieved parameters, such as pleural effusion, pericardial effusion, increased diameter of the IVC and its reduced collapse index, and diaphragmatic dysfunction. This new score, namely, integrated-LUS (i-LUS), whose maximum value reaches 40, has been applied to evaluate the diagnostic accuracy of thoracic ultrasound towards the critical issues of SARS-CoV-2 pneumonia and its impact on the targeted management of early in-hospital phase.

In this study, our i-LUS data, within one hour of admission to SEMD, for COVID positive patients with respiratory distress have been reported. Our data confirms those reported by other authors on the relationships between sex, age and outcome [24, 25]. There is a statistical difference (Table 1) for the groups' age when divided by sex (*p* = 0.005) and by outcome (*p* = 0.006). Most of the enrolled population was males (59.4% vs 40.6%; Fig. 3), females were older than males (79 years vs 71). Although the age difference lacks statistical significance, the findings suggest that women tended to survive infection more than men (43.1% deceased women vs 56.9% of deceased men).

Regarding comorbidities, which include hypertension, obesity, COPD, diabetes, cardiac and neurological disease (Table 1) the probability of survival in patients with less concomitant diseases is expressed as *p* < 0.05 between living and deceased.

The biochemical parameters indicative for inflammation, metabolism status, cellular death and lactate indices, obtained from blood samples collected at admission, were indirectly correlated with survival, lower in surviving patients at 30 days than in the deceased (Additional file 1: Table S1). PaO2/FiO2, was higher in surviving patients.

Our new i-LUS score was a valuable tool for quickly identifying and assessing early hospital stay for COVID-19 pulmonary disease. I-LUS was related to PaO2/FiO2. It was also shown that i-LUS and PaO2/FiO2 are related to the outcome: the mean score in the survivors was 15.9, while in the deceased was 18.6. In addition, there was a statistical difference between the groups of deceased and survivors, so the i-LUS score was a reliable indicator for assessment of lung injury severity, predicting disease outcome in COVID-19 positive patients, thus confirming the ability of i-LUS, as well as the arterial exchange indices, to predict the outcome of the patient with COVID-19 respiratory insufficiency.

Our i-LUS score data were in positive predictive correlation with the stratification of patients who needed NIV or oxygen therapy only. Moreover, the i-LUS score contributed significantly to the choice of the patient assignment unit. There was a significant difference between the i-LUS score of patients subsequently assigned to low intensity therapy (internal medicine/infectious diseases) and those transferred to the Intensive Care Unit (ICU; Fig. 5).

Finally, we also analysed the disease outcome data: the study shows that mortality at 30 days after patients’ admission was 45.5% (65 patients died and 78 survivors). Thirty-seven men (43%) died at follow-up, while 48 (57%) survived; 28 (48%) women died at follow-up, while 30 (52%) survived and on this population we calculated the Odds Ratio for death demonstrating a value of 1.08 (confidence interval 1.02–1.15) for a one-point increase for i-LUS.

As confirmed by other studies [26, 27], modified lung ultrasound protocols performed on COVID-19 patients increased the knowledge related to respiratory, cardiovascular and thromboembolic aspects compared to other imaging modalities. Pulmonary ultrasound patterns have been shown to improve monitoring of disease progression in critically ill COVID-19 patients [1, 10]. This information has been used to choose the better option—step by step—in ventilation modalities, as occurs in other COVID-like diseases states [21, 28–30]. With this new score and algorithm, it is possible to detect B lines and quantify the percentage of the pleural line associated with the lung diseases [31]. Direct involvement of cardiac function has been demonstrated in COVID-19 patients [32–34], and abnormal diaphragm function could be also present due to the prolonged ventilation patient effort, as reported for similar diseases [35–37]. Finally, due to the thrombotic tendency in COVID-19 patients, special attention needs to be paid to vascular ultrasound [38, 39]. For this reasons, the integrated evaluation of pulmonary, cardiac and diaphragmatic ultrasound, and IVC variation is important in SARS-CoV-2 infections. Our results are also coherent with those of other authors [40] that the addition of echocardiography in high-risk patients decreases the rate of misidentification of increased death risk. Our study evaluated i-LUS performed at admission to SEMD in COVID-19 positive patients with particular including criteria as mentioned before, demonstrating its predictive value on specific critical issues. The 12-areas approach in patients with COVID-19 infection, when implemented by cardiocirculatory and respiratory morphofunctional parameters measurable with ultrasound, could represent a rapid, accurate and feasible approach, in line with all prognostic indices, even in logistically difficult contexts.

## Limitations

The major diagnostic limitation is the low specificity of the signs detected using LUS in case of SARS-CoV-2 infection. It is related to the failure of recognizing pre-existing or overlapping lung pathologies, such as pulmonary edema, bacterial pneumonia, other forms of viral pneumonia and fibrosis lung disease, which may contribute to acute respiratory failure and influence the differential diagnosis [41]. Moreover, the study does not report comparative analysis between LUS and i-LUS with respect to the common pre-specified parameters, which will be the subject of our future investigations.

Interestingly, determining i-LUS does not require a skill level beyond the one required for the older tool (LUS score alone). The i-LUS score can be easily determined and used by medical doctors skilled for SEMD. The only difference is a longer implementation time than that for LUS.

## Conclusions

In conclusion, i-LUS can stratify COVID 19 patients with different degrees of lung disease, considering the heart status, volume status and diaphragm fatigue. With use of such “broaden and integrated LUS”, useful information for the appropriate management of patients based on their systemic disease severity, are acquired. This can help clinicians to choose the correct ventilatory support and adequate patient referral unit, as well as to contribute in disease outcome prediction. Further studies could contribute to define and standardize i-LUS as a valuable tool in the management of COVID-related conditions in all disease phases.

## Supplementary Information

**Additional file 1:** LUS_Covid.

**Additional file 2:** **Table S1.** Laboratory data and survival.

## Data Availability

All data and materials are available if requested.

## Abbreviations

CT: Computed Tomography,
LUS: Lung Ultrasound Score with 12 fields
SEMD: Sub-intensive Section of the Emergency Medicine Department
WFUMB: World Federation for Ultrasound in Medicine and Biology
NIV: Non-invasive Ventilation
i-LUS: Integrated Lung Ultrasound Score
