# Supplementary material for: Integrated lung ultrasound score for early clinical decision-making in patients with COVID-19: results and implications
Source: Ultrasound J. 2022 Jun 1;14:21. doi: 10.1186/s13089-022-00264-8 (PMC9156837; doi:10.1186/s13089-022-00264-8)
Supplement: Supplementary file 2 — Additional file 2: Table S1. Laboratory data and survival. [file 13089_2022_264_MOESM2_ESM.docx]

**Table S1 – Laboratory Data and Survival**

|  | **ALIVE** | **DEAD** | **P** |
| --- | --- | --- | --- |
| n | 78 | 65 |  |
| **LDH** | 289, [240.5, 410,5] | 367, [302,438] | 0.002 |
| **CPK** | 11.5, [63.5, 215,75] | 164, [79.75, 435.75] | 0.070 |
| **Mb** | 87, [55.5, 160.5] | 260, [152,673] | <0.001 |
| **TnI** | 11.5, [7, 23.1] | 53.9, [28.85, 145.95] | <0.001 |
| **PCR** | 64.1, [30.15, 114] | 109, [70.1, 165] | <0.001 |
| **DDimers** | 921.5, [529.5, 1240.5] | 1606.5, [705.25, 3655.5] | <0.001 |
| **Presepsine** | 517.5, [331.2, 673.25] | 891.5, [573.75, 1215.25] | <0.001 |
| **Lactates** | 1.1, [0.97, 1.6] | 1.7, [1.2, 2.7] | <0.001 |

Median; IQR;Wilcoxon rank sum test
